# Antigens Rv0310c and Rv1255c are promising novel biomarkers for the diagnosis of *Mycobacterium tuberculosis* infection

**DOI:** 10.1038/emi.2017.54

**Published:** 2017-07-12

**Authors:** Liulin Luo, Lin Zhu, Jun Yue, Jianping Liu, Guoyuan Liu, Xuelian Zhang, Honghai Wang, Ying Xu

**Affiliations:** 1 State Key Laboratory of Genetic Engineering, Institute of Genetics, Fudan University, Shanghai 200433, China; 2 Department of Clinical Laboratory Medicine, Shanghai Pulmonary Hospital, Tongji University School of Medicine, Shanghai 200433, China

**Keywords:** diagnostic, *M. tuberculosis*, pulmonary, sera, smear-positive, smear-negative

## Abstract

This study aimed to identify novel immunogenic epitopes from *Mycobacterium tuberculosis* (MTB) that could be used in tuberculosis (TB) diagnostics. To determine the diagnostic potential of mycobacterial antigens in serodiagnosis of TB, 256 patients were enrolled in a study and divided into two groups: 126 smear-positive pulmonary TB patients (SPPT) and 130 smear-negative pulmonary TB patients (SNPT); 152 bacillus Calmette-Guerin (BCG)-vaccinated healthy people were used as a control. Murine results showed that antigens Rv0310c-E from RD 8 and Rv1255c-E from RD 10 were strongly immunogenic to Th1 cells and induced a great humoral response. Receiver operating characteristic analysis indicated that Rv0310c-E (area under the curve (AUC): 0.800) and Rv1255c-E (AUC: 0.808) performed better than ESAT-6 (AUC: 0.665) and CFP-10 (AUC: 0.623) proteins but were comparable with Rv3425 (AUC: 0.788) protein in a human serum IgG analysis. Rv0310c-E demonstrated the highest diagnostic ability for the SPPT group (Youden index: 0.5602, sensitivity: 69.84%, specificity: 86.18%), while Rv1255c-E demonstrated the highest diagnostic ability for the SNPT group (Youden index: 0.5674, sensitivity: 73.84%, specificity: 82.89%). In addition, combination analysis found that antigen Rv0310c-E, coupled with the Rv3425 protein (Youden index: 0.6098, sensitivity: 87.30%, specificity: 73.68%) had the strongest performance for TB diagnostics of the SPPT group, and the single antigen Rv1255c-E was strongest for the SNPT group. These results suggest that antigens Rv0310c-E and Rv1255c-E are potential antigens for TB serodiagnostic tests, which may facilitate detection of MTB in smear-negative and smear-positive patients.

## INTRODUCTION

Tuberculosis (TB) caused by *Mycobacterium tuberculosis* (MTB) continues to be a major global health problem, with eight million new cases and two to three million deaths reported in 2015.^1^ The high morbidity and mortality rates associated with TB are driven by a number of contributing factors. These factors include infections caused by drug-resistant strains of TB, co-infection with human immunodeficiency virus (HIV) and inadequate treatment and prevention measures in low or lower-middle income nations.^1^ One of the major obstacles to controlling transmission of TB has been the lack of available diagnostic tools that are both accurate and inexpensive.^2^ With limited resources at the primary care level, inadequate diagnosis continues to be a major obstacle to global TB control in endemic countries.^3^

Bacteriological diagnosis of TB is directly confirmed by microscopic examination of acid-fast bacilli on sputum smears and culturing of MTB from patient samples.^4^ Most low and lower-middle income nations rely almost entirely on direct sputum smear microscopy for the diagnosis of pulmonary TB.^5^ The diagnostic accuracy of sputum smear microscopy is limited for adults and children, with ~57% of newly reported pulmonary TB cases presenting smear-positive pulmonary TB patients (SPPT).^4, 6^ In addition, smear-negative pulmonary TB patients (SNPT) represents between 30% and 60% of all pulmonary TB cases and is responsible for 10%–20% of all TB transmissions, although smear-positive patients are considered to be more infectious.^6, 7^ Therefore, delayed diagnosis in TB patients, especially in SNPT populations, may be an important cause of mortality and morbidity, and the proper diagnosis of SNPT cases is considered to be a top priority in TB control. Although the World Health Organization recommended diagnostic algorithms to detect SNPT in HIV-prevalent and resource-constrained settings, the sensitivity and negative likelihood ratio of the algorithms are poor in HIV-negative patients.^8^ There is a pressing need in the TB healthcare community for a simple and more efficient way to accurately detect MTB.

Serology has several advantages over other inexpensive diagnostic tools for identifying cases of TB patients. Serological tests detect antibodies raised against immunogenic mycobacterial antigens. These tests are easy to implement in the field and in lower-income nations because they are rapid and simple to perform.^9, 10^ One of the central criticisms of using serology to diagnose TB is that serological tests lack sensitivity (the ability to identify TB-positive patients, especially in cases of SNPT) and specificity (the ability to identify patients who are TB-negative) that are required for widespread clinical use.^11^ To enhance the sensitivity and specificity of serological-based assays, novel mycobacterial antigens must be identified that facilitate accurate diagnosis of both SPPT and SNPT patients.

Comparison of the pathogenic *M. tuberculosis* genome to the genomes of the vaccine *Mycobacterium bovis* bacillus Calmette-Guerin (BCG) strains identified 16 genomic regions of difference (RD), designated RD 1–RD 16 and nRD 18.^12, 13^ Genes within these regions encode potential antigens that could improve TB diagnosis or vaccine efficacy.^12^ Recently, two antigens from the RD 1 region (ESTA-6/CFP-10) were effectively used in the development of a novel skin test (C-Tb) that showed similar diagnostic sensitivity as the QuantiFERON-TB Gold In-Tube test in both HIV-positive and HIV-negative TB patient populations.^14^ In addition, other RD proteins, such as Rv1984c and Rv1985c from RD 2, Rv3425 from RD 11, Rv2645 from RD 13 and Rv3879 from RD 1, have shown potential as TB diagnostic tools.^15, 16, 17^

Despite growing evidence that the RD regions of the *M. tuberculosis* genome hold significant diagnostic potential, few studies have examined whether these epitopes can enhance the diagnostic accuracy of SPPT and SNPT patients, particularly the SNPT group. Previously, we analyzed specific RD regions of the *M. tuberculosis* genome in order to identify predicted CD8^+^ T-cell epitopes that could be further developed as vaccines or diagnostic tools.^18, 19^ In this study, we chose the Rv0310c protein from RD 8 and the Rv1255c protein from RD 10 based on our previous analysis and then evaluated the immunogenicity and immunoreactivity of these epitopes by murine experiments.^16, 18^ We further evaluated the sensitivity and specificity of these two epitopes in diagnosing SPPT and SNPT groups in order to determine which antigens are the most valuable for TB serodiagnosis. In addition, we combined different antigens for sera analysis to seek the best assembly of TB diagnostic.

## MATERIALS AND METHODS

### Study population

A total of 408 participants were recruited to participate in the study. The people that enrolled during the study period (January 2014–June 2015) were all collected from the Shanghai Pulmonary Hospital and analyzed. The study was approved by the Ethical Committee of Tongji University and Shanghai Pulmonary Hospital. All participants signed informed consent forms prior to being enrolled in the study.

Participants were divided into three groups: active pulmonary TB patients with a positive culture and a positive smear microscopy (SPPT), active pulmonary TB patients with a positive culture and a negative smear microscopy (SNPT) and BCG-vaccinated healthy controls.^20^ The criteria used for diagnosis of TB patients were: (i) interferon-γ release assay results were positive; (ii) presence of clinical symptoms including post meridiem mild fever, night sweat, expectoration and hemoptysis; (iii) computed tomography scan suggested TB infection and culture for mycobacterium TB was positive; (iv) anti-TB therapy was effective.^21, 22^ All patients underwent the initial diagnosis and treatment at the Shanghai Pulmonary Hospital, which is affiliated with Tongji University. TB patients who received chemotherapy for fewer than two weeks were included in our study. Controls were recruited from healthy people who had received a BCG vaccination during childhood. The criteria for the healthy control group included (i) no clinical signs or symptoms of TB; (ii) no TB contact history; (iii) normal chest X-ray or computed tomography scan.^23^ All of them received a physical examination and were confirmed to not have TB infection.

### Cloning, expression and purification of recombinant antigens

Of the 38 proteins selected from different RDs, Rv0310c from RD 8 and Rv1255c from RD 10 exhibited the best performance during serodiagnostic analysis (data not shown). Thus, these two proteins were chosen for further investigation in this study.^24^ The immunogenicity and antigenicity analysis of these two genes was performed using the TMHMM Server v. 2.0, SignalP 4.1 Server and Lasergene software.^19, 25^ The genes encoding the epitopes of the corresponding proteins were merged and biosynthesized (Invitrogen, Shanghai, China). Recombinant Rv3425, Rv3874 (EAST-6) and Rv3875 (CFP-10) proteins were constructed as previously described.^16^ Synthesized DNA sequences with *Nhe*I and *Xho*I restriction sites were cloned into pET30a, which places a six-histidine tag at the C terminus of each recombinant protein. The correct insert and orientation in all the constructions were verified by sequencing. Recombinant plasmids were used to transform *E. coli* BL21(DE3), and strains were grown in LB medium containing kanamycin (50 μg/mL). The expression of proteins was induced with 1 mM isopropyl-β-D-galactopyranoside at 37 °C for 4 h. The collected cells were lysed and then centrifuged at 13 000 rpm for 30 min, after which the supernatant was incubated with pre-equilibrated Ni Sepharose (GE Healthcare, Chicago, IL, USA) and gently agitated to maximize binding of the recombinant protein. Collected proteins were then refolded by using a 250- mL gradient of binding and washing buffers (20 mM Tris, pH 7.9, 100 mM imidazole) with the His-Bind Column (Novagen, Madison, WI, USA). At the end of the gradient, the desired protein was eluted with 20 mM Tris-HCl, pH 7.9, containing 200 mM imidazole. Proteins were quantified using the Bradford reagent (Bio-Rad, Hemel Hempstead, UK), and the presence of pure proteins was confirmed by separation on 15% SDS-PAGE. Recombinant proteins were also verified by western blot analysis using Anti-6X His tag antibody (Abcam, Shanghai, China).^9, 26, 27^ Endotoxin detection (Chinese Horseshoe Crab Reagent Manufactory, Xiamen, China), and the results are interpreted as previously described.^18^

### Murine assays: IFN-γ ELISPOT and cytokine measurement by sandwich ELISA and serum ELISA

C57BL/6 mice were injected subcutaneously with 50 μg of purified recombinant Rv0310c-E, Rv1255c-E and Rv3425 mixed with incomplete Freund’s adjuvant (IFA). Control group mice were injected with IFA only. Mice received two booster vaccinations at 2 weeks apart. At the end of the experiment, the mice were killed, and the spleens were removed. Lymphocytes were separated from other splenic cells using Lymphocyte-M density-gradient centrifugation (Cedar Lane Lab, Burlington, NC, USA). The isolated splenocytes were stimulated with purified recombinant protein (Rv0310c-E or Rv1255c-E or Rv3425, 10 μg/mL) for 36 h at 37 °C. To measure IFN-γ release by the stimulated splenocytes, a mouse IFN-γ ELISPOT assay kit (CT317-PR5; U-Cytech Biosciences, Utrecht, The Netherlands) was used according to the manufacturer’s instructions. The spots were visualized and counted using an immune-spot image analyzer.

The cytokine levels were also measured by sandwich ELISA. The lymphocytes were plated at a concentration of 2 × 10^6^ cells/well in 24-well plates in RPMI-1640 medium containing 10% fetal calf serum. The cells were stimulated with purified recombinant protein (Rv0310c-E, Rv1255c-E or Rv3425, 10 μg/mL) for 36 h at 37 °C. After stimulation, the supernatants were collected, and IFN-γ and TNF-α were analyzed using Cytokine ELISA MAX Set Deluxe kits (BioLegend, San Diego, CA, USA), following the manufacturer’s instructions.

Sera were collected from immunized mice prior to killing and stored at −80 °C. ELISA plates were coated overnight with 5 μg/mL of the corresponding protein, washed and blocked with phosphate-buffered saline (PBS) containing 1% bovine serum albumin (BSA). Serial dilutions of serum samples were added. Plates were incubated for 2 h at 37 °C and washed, followed by the addition of labeled IgG antibody (1:1000 dilution). The plates were incubated for 1 h at 37 °C, washed and developed with 0.1 M citrate-phosphate buffer that contained 1 mg/mL *o*-phenylenediamine and 0.03% hydrogen peroxide. Antibody titers are expressed as reciprocal endpoint titers. The reactions were stopped by addition of 2 M H_2_SO_4_ and were read on an ELISA plate reader at 492 nm.

### Antibody response screen of antigens by ELISA

Rv0310c-E and Rv1255c-E were evaluated for immunogenicity by screening human sera from clinically healthy individuals or patients with pulmonary TB for antibody responses. Antigens were coated in 96-well microplates at 400 ng/mL in 0.5 M carbonate–bicarbonate buffer overnight at 4 °C. The plates were washed three times with PBS that was supplemented with 0.05% Tween 20 (PBST) and blocked with PBST containing 1% BSA at 37 °C for 1 h. Blocked plates were incubated with human serum samples (1:50 dilution in PBS) at 37 °C for 2 h, washed again with PBST and incubated with peroxidase-conjugated anti-human IgG antibody (1:10 000 dilution in PBST-BSA; DAKO, Glostrup, Denmark) at 37 °C for 1 h. Plates were washed again with TBST three times, and the tetramethylbenzidine was added. The reaction was incubated at 37 °C for 10 min and stopped with 2.5 N H_2_SO_4_.^28^ Optical density was measured at 450 nm using a BioTek ELx808 plate reader (BioTek Instruments, Winooski, VT, USA). The TB-DOT kit that contains a 38-kDa antigen was also used as a comparison for measuring the level of the IgG antibody, and it was done according to the manufacturer’s recommendation (Shanghai Upper Bio-Tech Pharma Co. Ltd, Shanghai, China).

### Statistical analysis

Diagnostic values of IgG responses to recombinant proteins were examined by analysis of receiver operating characteristic (ROC) curves.^29^ The optimal operating point was determined via Youden’s index (YI) according to a method previously reported.^30^ The sensitivity and specificity of each operating point in the ROC curve and combined antigens were conditionally calculated using Excel, followed by calculation of YI at each point (YI=sensitivity+specificity−1). When evaluating the diagnostic performance of combined antigens in sera, any single positive was defined to be positive in combination, and the corresponding sensitivity and specificity were recorded. A one-way analysis of variance (ANOVA) was used to compare the mean protein concentration among different subjects. For comparisons of categorical data and continuous variables between different groups, a *χ*^2^-test (if the number was less than five, using Fisher’s exact test) and one-way ANOVA were used. All analyses were conducted using the SPSS software package (version 17.0; SPSS Inc., Chicago, IL, USA) and GraphPad Prism 5.0 (GraphPad Software, Inc., CA, USA). For all the tests, results with *P*<0.05 were considered significant.

## RESULTS

### Study population

A total of 408 participants were enrolled in our study, which consisted of 256 active pulmonary TB patients and 152 healthy control people. Out of these patients, 126 (49.2%) were smeared positive (SPPT) and 130 (50.8%) were smeared negative (SNPT). The characteristics of the study population are shown in Table 1.

The study subjects had a mean age of 41.72±17.85 years in SPPT, 44.43±18.07 years in SNPT and 43.21±13.24 in healthy control groups (*P*=0.414). There were no statistically significant differences between the three groups based on gender (*P*=0.975), CD4 levels (*P*=0.226) and IL-6 levels (*P*=0.030). As expected, there are some TB symptoms or comorbidities in patients, but these are absent in healthy people, and diabetes was more frequently found in the SPPT group than in the SNPT group.

One-way ANOVA analysis indicated that the differences in the BMI index and the serologic index, including the CD4/CD8 ratio and the ESR, CRP and TNF-α factors, were statistically significant between these groups. The BMI index and CD4/CD8 ratio, which relate nutrition and immunity conditions, were higher in the SNPT group than in the SPPT group, while the highest values were in BCG-immunized healthy people. Levels of inflammatory factors, such as ESR, CRP and TNF-α, were higher in SPPT patients than in SNPT patients, while the lowest was that of the controls. Detailed information is listed in Table 1.

### Generation of Rv0310c-E and Rv1255c-E antigens

**Figure 1:**
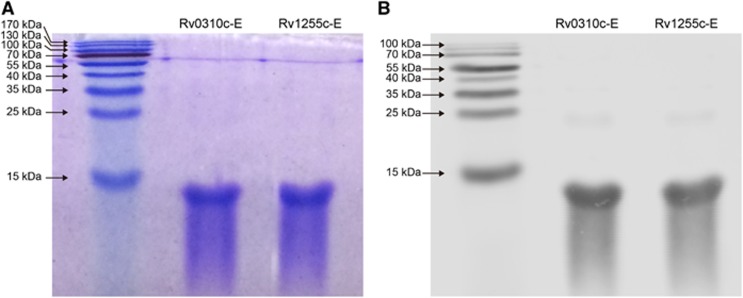
SDS-PAGE and western blot results of antigens Rv0310c-E and Rv1255c-E. (**A**) Electrophoresis analysis on a 15% SDS-PAGE gel stained with Coomassie blue; lane 1: marker; lanes 2 and 3: Rv0310c-E and Rv1255c-E recombinant antigens, corresponding to approximate molecular weights of 11 and 10 kDa, respectively. (**B**) Western blot analyses for antigens Rv0310c-E and Rv1255c-E. Lane 1: marker; lanes 2 and 3: Rv0310c-E and Rv1255c-E recombinant antigens.

Epitopes of antigens Rv0310c and Rv1255c were predicted. Fragments containing dominant epitopes without a transmembrane helix or a signal peptide in the N terminus but with good antigenicity and hydrophilicity were chosen. Antigen Rv0310c contains six epitopes that consist of amino acids (AA) 8–17, 29–40, 43–50, 120–132, 135–143 and 154–163, while Rv1255c contains three epitopes that consist of 114–129, 139–155 and 166–177 AA. Detailed peptide information is present in Table 2. All epitopes were separately merged for each antigen and used for cloning, purification and overexpression, and the corresponding fragments were then named Rv0310c-E and Rv1255c-E, respectively. Control proteins Rv3425, EAST-6 and CFP-10 were constructed according to our previously described methods.^16, 19^ Purified recombinant proteins Rv0310c-E and Rv1255c-E were fractionated by electrophoresis on a 15% polyacrylamide gel. Bands corresponding to 11-kDa and 10-kDa proteins were observed on a gel stained with Coomassie brilliant blue dye (Figure 1A) and the anti-His antibody (Figure 1B). Low endotoxin concentrations (<0.9 EU/mg) were typically observed and subsequently used for animal experiments and clinical evaluation.

### Proteins Rv0310c-E and Rv1255c-E evoked strong immune responses in mice

**Figure 2:**
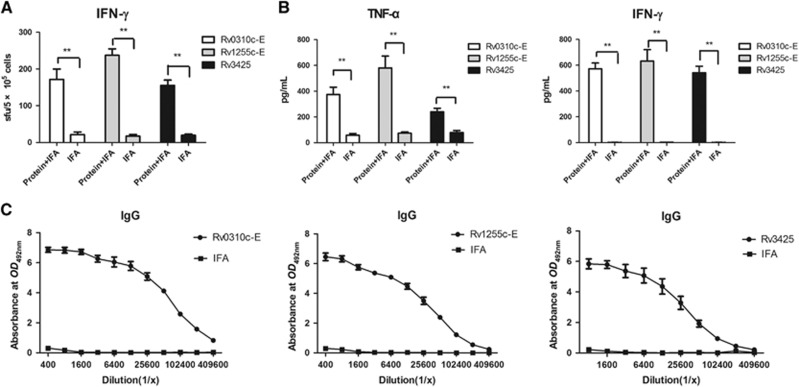
Rv0310c-E and Rv1255c-E proteins evoked strong immune responses in mice. The cellular immune response and antibody response in mice immunized with protein Rv0310c-E, Rv1255c-E or Rv3425 in IFA and IFA alone are shown. Splenocytes (5 × 10^5^ cells/well) were stimulated with Rv0310c-E or Rv1255c-E or Rv3425 (10 μg/mL) for 36 h at 37 °C and 5% CO_2_. IFN-γ activities in suspensions of single splenocytes were measured in an ELISPOT assay. (**A**) The cell supernatants were collected, and IFN-γ and TNF-α levels were measured by sandwich ELISA. (**B**) Serum samples were collected and analyzed by ELISA for the presence of anti-Rv0310c, anti-Rv1255c or anti-Rv3425 IgG. (**C**) The data are representative of two separate experiments. **P <0.05 vs. IFA.

To further validate the immunogenicity of the two novel epitopes in comparison to Rv3425, mice were immunized with recombinant Rv0310c-E, Rv1255c-E and Rv3425. Splenocytes from immunized mice were stimulated with the recombinant antigens, and T-cell activation was measured by IFN-γ production (Figure 2A). The IFN-γ responses by T cells that were exposed to either Rv0310c-E or Rv1255c-E antigens exceeded (*P*<0.005) the response by the adjuvant control groups. Responses in the mice that were immunized with Rv1255c-E were higher than the responses of mice immunized with either Rv0310c-E or Rv3425 (*P*<0.05). The secretion of IFN-γ and TNF-α by mouse spleen cells was detected by sandwich ELISA, as shown in Figure 2B. Mice immunized with Rv1255c-E showed an enhanced release of TNF-α compared with the levels of release by the group immunized with Rv0310c-E and Rv3425 (*P*<0.05). The concentration of IFN-γ in mice immunized with Rv1255c-E was higher than the responses of mice immunized with Rv0310c-E and Rv3425, which was consistent with the data presented in Figure 2A. These results demonstrate that Rv0310c-E and Rv1255c-E could evoke high Th1 responses and that Rv1255c-E may be more immunogenic than Rv0310c-E and Rv3425.

To evaluate B-cell-mediated immune responses against Rv0310c-E, Rv1255c-E and Rv3425 proteins, IgG antibody levels were measured in sera taken from mice that were immunized with these antigens. Compared with the IFA control group, mice vaccinated with Rv0310c-E, Rv1255c-E or Rv3425 proteins produced higher levels of IgG antibodies (Figure 2C). However, IgG antibody titers from Rv0310c-E- or Rv1255c-E-immunized mice were higher than the IgG titers from Rv3425-immunized mice (*P*<0.05). Thus, Rv0310c-E and Rv1255c-E induced a slightly stronger humoral response than Rv3425.

### Clinical evaluation of Rv0310c-E and Rv1255c-E recombinant proteins in human sera

**Figure 3:**
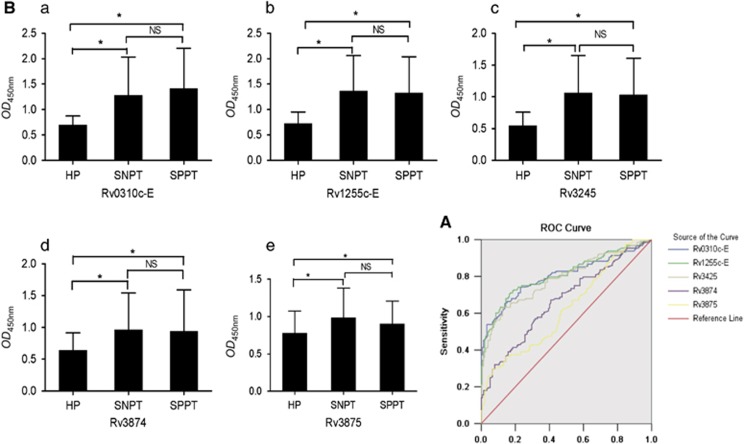
ROC analysis of five antigens and comparison between SPPT, SNPT and healthy groups. (**A**) ROC curves to discriminate among antibody responses against five mycobacterial antigens. Serum samples were obtained from 256 active TB patients and 152 BCG-vaccinated healthy people controls. Antibody responses were measured by ELISA. The area under the ROC curve (AUC) for each antigen is listed as follows: Rv0310c-E AUC: 0.800 (95% CI, 0.759–0.842). Rv1255c-E AUC: 0.808 (95% CI, 0.767–0.849). Rv3425 AUC: 0.788 (95% CI, 0.745–0.831). Rv3874 AUC: 0.665 (95% CI, 0.613–0.718). Rv3875 AUC: 0.623 (95% CI, 0.568–0.677). (**B**) The difference in five antigens between TB patients and controls. Statistically significant differences were found between patients and controls, but none were found in SPPT and SNPT.

To evaluate the antigenicity of proteins Rv0310c-E and Rv1255c-E, human sera was obtained from 408 participants, and circulating IgG titers against these epitopes were measured. Three well-known immune-dominant antigens of *M. tuberculosis*, ESAT-6, CFP-10 and Rv3425 were also tested as positive controls. The ROC curves (Figure 3A) show that the antigenicity of Rv0310c-E and Rv1255c-E was higher than that of ESAT-6 and CFP-10 but comparable with that of Rv3245. When differentiating between active pulmonary TB patients and healthy controls, the ROC curves for Rv0310c-E and Rv1255c-E had an area under the curve (AUC) of 0.800 (95% CI, 0.759–0.842) and 0.808 (95% CI, 0.767–0.849), respectively. These values exceeded those of ESAT-6 (AUC: 0.665, 95% CI, 0.613–0.718) and CFP-10 (AUC: 0.623, 95% CI, 0.568–0.677), but were comparable with that of Rv3425 (AUC: 0.788, 95% CI, 0.745–0.831). We also compared these five antigens between patient groups and healthy controls. All of them showed statistically significant differences between patients and controls, but none were found in SPPT and SNPT groups (Figure 3B).

The optimal operating point for each antigen at the maximum value of YI was calculated from the ROC analysis, and the corresponding sensitivity and specificity were determined.^30^ The overall diagnostic performances of individual antigens for active TB are shown in Table 3. The best performing antigen in the SPPT group was Rv0310c-E (YI: 0.5602), while it was Rv1255c-E (YI: 0.5674) in the SNPT group. Based on the highest YI (the larger YI, the better diagnostic performance), the sensitivity and specificity of Rv0310c-E in SPPT group were 69.84% and 86.18%, respectively. Similarly, the sensitivity and specificity of Rv1255c-E in SNPT group were 73.84% and 82.89%.

To better evaluate the humoral antigenic features of antigens Rv0310c-E and Rv1255c-E, the IgG response was compared with the commercial TB-DOT kit, which contains the 38-kDa protein for the diagnosis of active pulmonary TB patients. The YI values of TB-DOT in the SPPT and SNPT groups were 0.3575 and 0.2595. Based on the specificity of 92.10%, the sensitivity of this kit in the SPPT and SNPT groups were 43.65% and 33.84%, respectively (data not shown). These results showed that both Rv0310c-E and Rv1255c-E were highly immunogenic proteins and that all three proteins exhibited good performance in the sera analysis of TB patients.

### Serodiagnostic performance of antigen combinations

To further assess the antigens’ serodiagnostic performance in TB patients, we combined different antigens to get the best result. During the combination analysis, a sample was found to be positive when any assay was considered to be positive.

Among the two-antigen combinations, the Rv0310c-E and Rv3425 pairing showed the highest diagnostic ability (YI: 0.6098) in the SPPT group, while the combination of Rv1255c-E and Rv3425 exhibited the maximal YI (0.5674) in the SNPT group. The sensitivity and specificity in the SPPT group were 87.30% and 73.68%, respectively, while they were 84.61% and 70.39% in SNPT group, respectively (Table 3). For combinations of three antigens, the Rv0310c-E, Rv3425 and CFP-10 trio performed the best (YI: 0.5823) in the SPPT group, while the Rv1255c-E, Rv3425 and the CFP-10 combination were better in the SNPT group (YI: 0.526). The corresponding sensitivity and specificity in SPPT groups were 90.47% and 67.76%, respectively, while they were 86.15% and 66.44% in the SNPT group, respectively (Table 3). However, the more antigens that are used combination for a diagnostic, the lower the resulting specificity would be. When we combined four antigens, the best results were obtained by using Rv0310c-E, Rv1255c-E, Rv3425 and CFP-10 together, in both the SPPT and SNPT groups. The corresponding sensitivities were 92.06% (SPPT), 86.15% (SNPT) and the specificity was 55.92% (Table 3). This combination would increase the detection sensitivity but at the cost of specificity during the analysis.

In summary, based on analysis using single or combinations of antigens, the best result in the SPPT group was the combination of the Rv0310c-E and Rv3425 antigens (YI: 0.6098, sensitivity: 87.3%, specificity: 73.68%), while that in the SNPT group was the single antigen Rv1255c-E (YI: 0.5674, sensitivity: 73.84%, specificity: 82.89%).

## DISCUSSION

TB is an infectious bacterial disease that is caused by MTB. The number of patients with pulmonary TB is increasing rapidly in endemic areas that demand fast and accurate diagnosis of these patients, which is both challenging and relevant to the global public health approach to TB control, especially for smear-negative pulmonary TB patients.^6, 31, 32^ It is especially difficult to diagnose pediatric patients that have pulmonary TB because children may struggle to provide enough sputum or expectorate for an adequate smear.^4^ In our study, SNPT cases represented 50.8% of all pulmonary TB cases that were reported in the study, indicating that the prevalence of SNPT was higher than that of SPPT in our Chinese patient population.

Several inexpensive diagnostic tools exist for the detection of MTB, including clinical manifestations, chest X-rays and tuberculin skin tests, but these methods may not be sensitive enough to detect the majority of SNPT cases.^33, 34^ Patients with presumptive HIV-associated SNPT can be diagnosed using the automated GeneXpert system, which has been suggested as a replacement for smear microscopy in low-income nations if assay costs are offset by international funding.^35^ Thus, the rapid, economic and accurate point-of-care tools for TB diagnosis are urgently required. Considering these challenges, new serodiagnostic tools are needed to provide both sensitive and specific detection of TB patients, especially in SNPT groups but at a significantly reduced cost.

On the basis of our laboratory’s previous results, 38 proteins were chosen from different RDs, and our findings indicated that proteins Rv0310c from RD 8 and Rv1255c from RD 10 exhibited the best performance during serodiagnostic analysis. Thus, these two proteins were chosen for further investigation in this study.^24^ Both proteins do not appear to share homology with known virulence factors. Rv0310c is a hypothetical protein in the *M. tuberculosis* H37Rv genome (Gene ID 886570), and it has nine human leukocyte antigen-binding positions, while Rv1255c shares homology with several helix-turn-helix-type DNA-binding transcriptional regulators (Gene ID 887068), and it has 14 human leukocyte antigen-binding positions.^36^ There are some advantages in using antigen fragments over the whole protein; fragments consisting of immunodominant epitopes have superior or comparable binding affinities for antibodies and result in the removal of redundant sequences that would block exposure of the immunodominant epitopes.^37, 38^ This approach has been used successfully for the mycobacterial antigens ESAT-6 and CFP-10, which were fused together in order to significantly increase T-cell activation, IFN-γ production and diagnostic sensitivity.^38, 39, 40^ By virtue of bioinformatics analysis, six epitopes of Rv0310c and three epitopes of Rv1255c were chosen for further research. It is necessary to evaluate the immunogenicity and immunoreactivity of these fragments before using them in a diagnostic application. Thus, we prioritized and performed the murine experiments in this work.

In the mouse model experiments, we evaluated the immune responses to Rv0310c-E and Rv1255c-E antigens in comparison with the positive control protein Rv3425 that we previously reported.^16, 18^ Antigen Rv1255c-E has evoked the greatest immuno-response among these proteins. Immunization with Rv1255c-E induced stronger antigen-specific IFN-γ activities than those in the groups immunized with either Rv0310c-E or Rv3425. In addition, mice immunized with Rv1255c-E showed an enhanced release of TNF-α and IFN-γ compared with the levels of release in the groups immunized with Rv0310c-E and Rv3425 (*P*<0.05). Moreover, the antigen-specific IgG for mice immunized with Rv1255c-E and Rv0310c-E was also higher than that for Rv3425-immunized group. These results indicated that antigens Rv0310c-E and Rv1255c-E were not only strongly immunogenic to Th1 cells, but also induced a great humoral response. Proteins Rv3425 and Rv2659 from RD 11 and Rv2645 from RD 13 were recognized as TB-specific T antigens, while at the same time they could cause a prominent humoral response.^16, 17, 27^ Here we continue to evaluate the humoral response of antigens in TB patients and BCG-vaccinated healthy controls.

Proteins encoded by RD regions of *M. tuberculosis* have been demonstrated to be important in bacterial virulence, vaccine development and diagnostics.^19, 41^ Proteins, such as Rv1985c, Rv3871, Rv3876 and Rv3879, that originated from RD 2 and 1 were demonstrated to be promising antigens in TB diagnostics, while Rv2659c was more suitable for diagnosis in latent TB patients.^23, 27, 30^ In our study, the results of anti-Rv0310c-E, based on RD 8, and anti-Rv1255c-E, based on RD 10, IgG titers were significantly higher in TB patients than controls. Using ROC analysis, we found that anti-Rv0310c-E and anti-Rv1255c-E IgG antibodies exhibited similar AUC values. The AUC values of Rv0310c-E and Rv1255c-E exceeded the values of two proteins that are currently used in the clinical T-SPOT.TB assays (ESAT-6 and CFP-10), but were comparable values obtained with Rv3425. We also analyzed antigen performances in different groups and found that levels of all five proteins were significantly different between TB patients and healthy controls, but none were found in the SPPT and SNPT groups. Further analysis indicated that Rv0310c-E was the best antigen in SPPT groups, while the best in SNPT groups was Rv1255c-E, and the performance of both of these proteins were superior to that of the positive control 38-kDa protein. Since the SNPT group is a relatively high fraction of TB patients, it seems likely to be more responsible for the disease’s transmission.^7, 42^ The Rv1255c-E antigen may be a particularly promising antigen for diagnostics of SNPT, and this diagnostic would be greatly beneficial to public health. Our results were also in accordance with those of previously reported antigens.^23, 27, 30^ However, single antigens were still insufficient to meet the clinical diagnostic requirements. Thus, antigen combinations are encouraged for use in TB diagnostic.

The combination of different antigens, including epitopes, proteins, glycolipids and other kinds of Ig classes, were used so that their diagnostic ability could be evaluated. The combination of Rv3871, Rv3876 and Rv3879 demonstrated good performance in SNPT patients.^30^ Fusion protein epitopes, such as 38F, 64F, TB16.3-echA1, tn2FbpC1-tnPstS1 and tnHSP-tn1FbpC1, showed improved diagnostic ability for different TB patients.^37, 38, 40^ Other protein chips or protein combinations also exhibited favorable diagnostic ability for pulmonary and extrapulmonary TB patients.^43, 44, 45^ During our combination analysis, a sample found to be positive for any assay was considered to be positive, and the best diagnostic performance that was obtained in the SPPT group was the assembly of the two antigens Rv0310c-E and Rv3425; use of three or more antigens would reduce the diagnostic ability. In the SNPT group, the best performance was that of the signal antigen Rv1255c-E; other combinations exhibited greatly reduced the diagnostic ability. Our research would enrich the diagnostic performance of antigens by combining different epitopes.

Although our present study has found promising diagnostic antigens (Rv0310c-E in the SPPT group and Rv1255c-E in the SNPT group), there is still a large gap that must be bridged to satisfy the clinical diagnostic demands. There are several limitations to the present study that must be mentioned. First, during our cohort study, no extrapulmonary TB patients or controls that had other diseases were enrolled. Second, other Ig class members, such as IgM and IgA, were also reported with MTB infections in early serum diagnostics; however, we did not assess these proteins in this study. They should be explored in our next study.^46, 47^ In addition, epitope combinations that originated from within only a single gene were tested, although combinations from different genes should also be analyzed. Future studies will combine the epitopes of Rv0310c-E and Rv1255c-E or even other known proteins such as Rv3425, the 38-kDa protein and MTP64 to create fusion antigens, which will be tested to see if further combinations can contribute to increased diagnostic ability.

In conclusion, we have identified two diagnostic mycobacterial antigens (Rv0310c-E and Rv1255c-E) that can be used in SPPT and SNPT groups. These antigens do not share homology with known mycobacterial virulence factors, but they induce both humoral and cell-mediated immune responses in mice. Although our study is foundational for further investigations targeting clinical applications, both identified proteins demonstrate potential as sensitive and specific serodiagnostic tools that could provide serum antigen candidates to easily and accurately detect MTB in smear-negative and smear-positive patients.

## Figures and Tables

**Table 1:** Clinical characteristics of study participants by classification group

**Characteristic**	**SPPT (*n*=126)**	**SNPT (*n*=130)**	**HP (*****n*****=152)**	***P***
Age (years)	41.72±17.85	44.43±18.07	43.21±13.24	0.414
Male/Female (*n*)	59/67	61/69	73/79	0.975
BMI index (kg/m^2^)	19.86±2.37	20.61±2.38	23.16±3.86	<0.001

*Comorbidities*				
Diabetes (*n*)	22	7	0	<0.001

*TB symptom*				
Mild fever (*n*)	53	54	0	<0.001
Night sweat (*n*)	12	14	0	<0.001
Expectoration (*n*)	92	80	0	<0.001
Hemoptysis (*n*)	12	16	0	<0.001

*Serologic index*				
CD4 (%)	37.88±45.94	35.53±10.29	41.00±8.52	0.226
CD8 (%)	29.71±26.58	23.94±11.30	21.56±6.58	<0.001
CD4/CD8	1.55±1.20	1.90±1.23	2.01±0.60	0.001
ESR (mm/h)	56.65±32.91	33.88±28.38	21.07±16.28	<0.001
CRP (mg/L)	53.14±47.19	29.28±35.91	5.02±2.58	<0.001
IL-6 (ng/L)	49.50±36.80	48.89±111.04	31.14±10.12	0.030
TNF-a (pg/mL)	58.72±52.98	51.24±32.98	42.90±18.67	0.002

Statistics: age (years): mean±sd; BMI index: mean±sd. No significant differences in age or gender were found between healthy controls and TB patients (*χ*^2^-test and one-way ANOVA, *P*>0.05). The BMI index in TB patients is lower than in the BCG-immunized healthy people, and the statistical difference is significant (*χ*^2^-test and one-way ANOVA, *P*<0.01).

**Table 2:** Detailed peptide sequences of Rv0310c and Rv1255c proteins

**Peptide location**	**Peptide sequence**
P1: Rv0310c(8–17)	TPGDPADIAA
P2: Rv0310c(29–40)	LDTKHWDDFTDT
P3: Rv0310c(43–50)	EDVTGDYG
P4: Rv0310c(120–132)	YHDQYRRTTDGWR
P5: Rv0310c(135–143)	ATGYDRTYE
P6: Rv0310c(154–163)	NIRPGRALAD
P1: Rv1255c(114–129)	TTRPPIGGEMAGRSEV
P2: Rv1255c(139–155)	NSLGPDDPTTVERRARW
P3: Rv1255c(166–177)	FPGRDEADERAM

Annotations: peptide sequences of proteins Rv0310c and Rv1255c were biosynthesized, merged and optimized for expression.

**Table 3:** Diagnostic performance of antigen combinations

**Combined antigens**	**Sensitivity (SPPT)**	**PPV (SPPT)**	**Youden (SPPT)**	**Sensitivity (SNPT)**	**PPV (SNPT)**	**Youden (SNPT)**	**Specificity (HP)**	**NPV (SPPT)**	**NPV (SNPT)**
A	0.6984	0.8073	0.5603	0.6077	0.7900	0.4695	0.8618	0.7751	0.7198
B	0.6825	0.7679	0.5115	0.7385	0.7869	0.5674	0.8289	0.7590	0.7875
A+B	0.8254	0.7027	0.5359	0.7692	0.6944	0.4798	0.7105	0.8308	0.7826
A+C	0.8730	0.7333	0.6099	0.7692	0.7143	0.5061	0.7368	0.8750	0.7887
A+E	0.7778	0.7656	0.5804	0.7154	0.7561	0.5180	0.8026	0.8133	0.7673
A+B+C	0.9206	0.6516	0.5127	0.8461	0.6395	0.4382	0.5921	0.9	0.8181
A+B+E	0.8253	0.6797	0.503	0.7846	0.6754	0.4622	0.6776	0.824	0.7862
A+C+E	0.9047	0.6993	0.5823	0.8307	0.6878	0.5084	0.6776	0.8956	0.824
B+C+E	0.8571	0.6792	0.5216	0.8615	0.6871	0.526	0.6644	0.8487	0.8487

Abbreviations: Rv0310c-E, A; Rv1255c-E, B; Rv3425, C; Rv3875, E; smear-positive TB patient, SPPT; smear-negative TB patient, SNPT; healthy control population, HP; positive predictive value, PPV; negative predictive value, NPV.

Youden index=sensitivity+spectivity-1.
